# Quantitative profiling brain proteomes revealed mitochondrial dysfunction in Alzheimer’s disease

**DOI:** 10.1186/s13041-019-0430-y

**Published:** 2019-01-28

**Authors:** Sunil S Adav, Jung Eun Park, Siu Kwan Sze

**Affiliations:** 1 School of Biological Sciences, Division of Structural Biology and Biochemistry, Nanyang Technological University, 60 Nanyang Drive, Singapore, 637551 Singapore; 2 Singapore Phenome Centre, Lee Kong Chian School of Medicine, Nanyang Technological University, 59 Nanyang Drive, Singapore, 636921 Singapore

**Keywords:** Alzheimer’s disease, Neurodegenerative diseases, Mitochondrial dysfunction, Mitochondriome, Complex I, Proteomics, iTRAQ

## Abstract

Mitochondrial dysfunction is a key feature in both aging and neurodegenerative diseases including Alzheimer’s disease (AD), but the molecular signature that distinguishes pathological changes in the AD from healthy aging in the brain mitochondria remain poorly understood. In order to unveil AD specific mitochondrial dysfunctions, this study adopted a discovery-driven approach with isobaric tag for relative and absolute quantitation (iTRAQ) and label-free quantitative proteomics, and profiled the mitochondrial proteomes in human brain tissues of healthy and AD individuals. LC-MS/MS-based iTRAQ quantitative proteomics approach revealed differentially altered mitochondriomes that distinguished the AD’s pathophysiology-induced from aging-associated changes. Our results showed that dysregulated mitochondrial complexes including electron transport chain (ETC) and ATP-synthase are the potential driver for pathology of the AD. The iTRAQ results were cross-validated with independent label-free quantitative proteomics experiments to confirm that the subunit of electron transport chain complex I, particularly NDUFA4 and NDUFA9 were altered in AD patients, suggesting destabilization of the junction between membrane and matrix arms of mitochondrial complex I impacted the mitochondrial functions in the AD. iTRAQ quantitative proteomics of brain mitochondriomes revealed disparity in healthy aging and age-dependent AD.

**Electronic supplementary material:**

The online version of this article (10.1186/s13041-019-0430-y) contains supplementary material, which is available to authorized users.

## Introduction

Alzheimer’s disease (AD) is an age-dependent multifactorial neurodegenerative disorder with wide clinical heterogeneity which progressively impairs cognitive and memory functions. Aging is the greatest risk factor for neurodegenerative diseases including AD, Parkinson’s disease (PD) Huntington’s disease (HD) and amyotrophic lateral sclerosis (ALS) [1–3]. According to the free radical theory of aging, increased mitochondrial reactive oxygen species (ROS) with age causes mutations in mtDNA and damage to mitochondrial components resulting in cellular senescence [4]. While, amyloid cascade hypothesis of AD assumes that mutation in amyloid precursor protein (APP) causes increased production or decreased disposal of Aβ, leading its accumulation that impedes the mitochondrial function [5]. This hypothesis and an accumulated literature contemplate the mitochondria as a crucial organelle in numerous mechanisms implicated in aging and multiple neurodegenerative diseases including AD, PD, HD, and ALS [5–7].

Most of the ATP of a cell is produced through oxidative phosphorylation (OXPHOS) in mitochondria which is driven by the electron transport chain (ETC). OXPHOS is composed of four respiratory complexes (RC), called complex I (NADH-ubiquinone oxidoreductase), complex II (succinate: ubiquinone oxidoreductase), complex III (ubiquinol-cytochrome c reductase), and complex IV (cytochrome c oxidase); the electron carriers ubiquinone (UQ or CoQ) and cytochrome c (cyt c); and the ATP synthase (complex V) [8]. Each complex composes of several subunits, i.e. complexes I, II, III, IV, and V respectively compose of 45, 4, 11, 13 and 16 subunits. The involvement of several complexes and their numerous subunits makes mitochondrial biogenesis as an extremely complex process and each subunit is thought to be involved in a dynamic balance in the composition of the mitochondriome that determines mitochondrial function [9]. The functional defect in any single subunit can cause mitochondrial dysfunction, e.g. mutation in NDUFA2 causes reduced activity and disturbed assembly of mitochondrial complex I [8, 10]; NDUFA4 mutation results in dysfunction of a cytochrome c oxidase subunit [11]; while mutation in the subunit NDUFA13 leads to instability of mitochondrial complex I that affects motor nerve control by the brain [12]. Restated, each subunit of each mitochondrial complex remains critical for normal mitochondrial function, but, very rarely, attempts have been made to explore regulatory profile of complexes and their subunits in aging and AD pathology.

Mitochondria play a major role in energy production, calcium regulation, maintenance of plasma membrane potential, protein folding by chaperones, axonal and dendritic transport, and the release and re-uptake of neurotransmitters at synapses [13–15]. The regulatory pathways including intermediate metabolism, steroid metabolism, amino acid biosynthesis, fatty acid oxidation, apoptosis etc. take place in mitochondria. Consequently, mitochondrial dysfunction can have grave consequences on these processes and defects in energy production [16]. Morphological changes such as abnormally rounded mitochondria [17], decrease in the number of mitochondria [18], decrease in mtDNA copy number [19, 20], decrease in glutathione and mitochondrial cytochrome aa3 [21], decreased NADH-cytochrome c reductase activity in mitochondria isolated from rat and mice liver, brain, heart, and kidney upon aging have been documented [22, 23]. An abnormal accumulation of amyloid precursor protein (APP) across mitochondrial import channels leads to mitochondrial dysfunction and play major role in AD pathology [24]. The abnormalities of tau and its association with mitochondrial dysfunction and impaired axonal transport of mitochondria in AD is reviewed by Reddy [25]. Mitochondria play a key role in ageing-related neurodegenerative diseases; therefore, the therapies targeting basic mitochondrial processes are most promising [26]. An accumulation of Aβ in mitochondria leads to oxidative damage [5, 6, 27] and disrupted electron transport chain (ETC). As mitochondrial ETC and ATP-synthase are composed of numerous subunits, morphological mitochondrial characteristics and enzymatic activities assay remain limited in profiling subunit-specific abundances, their regulations and specific cause of mitochondrial dysfunction.

According to Swerdlow’s [2, 28] “mitochondrial cascade hypothesis”, brain aging and AD are mechanistically related. Although, aging and AD are mechanistically related, only some elderly people have an AD. We assume that there could be several alterations in mitochondriome and differences in mitochondrial processes that discriminate aging and age-dependent AD pathology. Proteomic technology has the potential to explore regulatory profile of each subunit of the mitochondrial complex. Therefore, this study adopted discovery-driven quantitative proteomics approach coupled with electrostatic repulsion-hydrophilic interaction chromatography (ERLIC) shotgun proteomics to explore quantitative abundances of brain proteome of the AD and their respective controls through isobaric tags for relative and absolute quantitation (iTRAQ) by nano-LC-MS/MS. An iTRAQ quantitative brain mitochondrial proteome regulation in AD patient made us strive to isolate purified brain mitochondria and further explored mitochondriome by label-free proteomics. Our both quantitative and label-free proteomic data revealed differential altered protein profile of electron transport chain and mitochondrial proteins in AD pathology and demonstrated destabilization of the junction between membrane and matrix arms of complex I.

## Results

### Characteristics and quantitative profile of brain mitochondrial proteins

We explored the human brain proteome of medial frontal gyrus of 68.0 ± 2.0 and 83.8 ± 3.5 years old women AD patients with their age-matched non-demented controls using iTRAQ quantitative mass spectrometric technique. We termed 68.0 ± 2.0 years AD patients as an early-onset AD (iTRAQ ratio: 115/114) and 83.8 ± 3.5 years AD patients as a late-onset AD (iTRAQ ratio:117/116). Further, ratios of (116/114) can be considered as protein alterations in healthy aging individuals, while 117/115 as an aging AD. Braak staging as devised by Braak [29] to indicate disease progression was level 6 in early-onset AD patients, while it was 5.4 in the late-onset AD. The amyloid staging was graded C in both early and late onset AD patients and their corresponding grading was A or B in their controls. The other clinical characteristics incluing postmortal duration (PMD) are listed in Table 1.

**Table 1:** Clinical characteristics of the AD patient. PMD: post mortem delay, ApoE: Apolipoprotein E

iTRAQ Label used	Age (Years)	Gender	BRAAK	Amyloid	PMD	ApoE	Diagnosis	Region
114	67.0 ± 3.0	Female	1.0 ± 1.0	A	5.7 ± 0.3	32.5 ± 0.5	Non-demented control	medial frontal gyrus
115	68.0 ± 2.0	Female	6.0 ± 0.0	C	5.2 ± 0.0	38 ± 5.0	Alzheimer’s disease	medial frontal gyrus
116	84.0 ± 3.2	Female	1.6 ± 0.8	B	6.3 ± 2.4	32.5 ± 0.5	Non-demented control	medial frontal gyrus
117	83.8 ± 3.5	Female	5.4 ± 4.9	C	6.0 ± 1.5	37 ± 4.9	Alzheimer’s disease	medial frontal gyrus

**Fig. 1:**
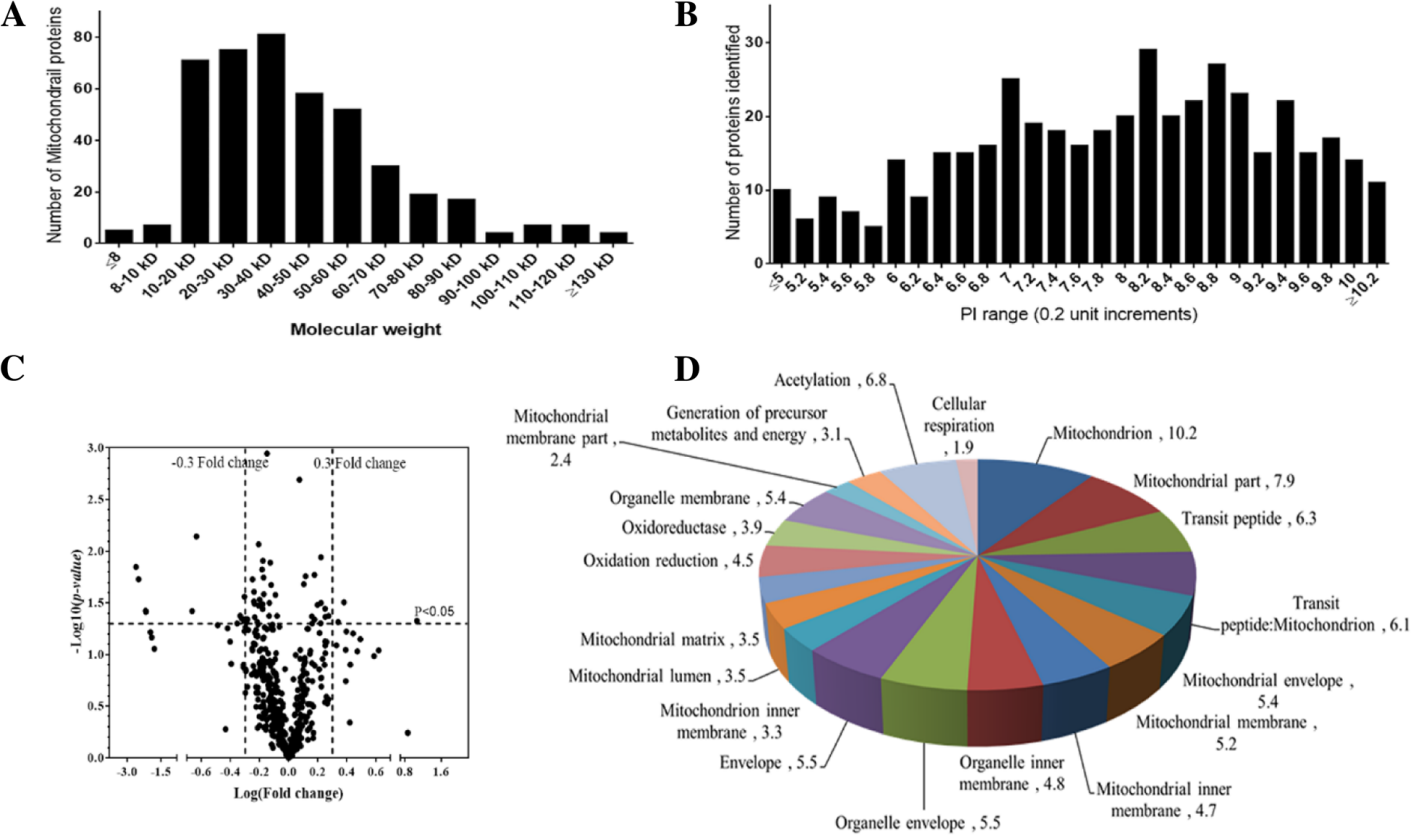
Brain mitochondrial proteins properties, regulation and localization. **a** Molecular weight distribution of iTRAQ quantified brain mitochondrial proteins, **b** pI distribution of iTRAQ quantified brain mitochondrial proteins, **c** Volcano plot of the log_10_(*p*-values) as a function of log_2_(protein fold change). The statistical significance (*p*-value in a − log_10_ scale) is plotted as a function of the protein fold change (in a log_2_ scale), **d** Cellular localization of mitochondrial proteins as determined by gene ontology

We acquired a total of 284,352 ± 4564 spectra from three iTRAQ replicates over a total of 60 LC-MS/MS runs. Using PD with set criteria of high peptide confidence, we identified 4814 ± 68 proteins (Additional file 1: Table S1) with 679,500 ± 7500 peptides. We narrow down the protein list to 3015 ± 75 proteins (Additional file 2: Table S2) by considering proteins identified in at least two MS run and with more than two peptides. We subjected iTRAQ-quantified brain proteome data to Mitocarta database and extracted mitochondrial proteins. Based on mitocarta database, a substantial portion (434 proteins, Additional file 3: Table S3) of brain proteome were of mitochondrial origin. Based on pI histogram, integral membrane proteins clusters around pI 8.5–9.0, whereas cytosolic proteins clustered around pI values of 5–6. In eukaryotes, there was also a third cluster with pI values around 7.0. The iTRAQ quantified mitochondrial proteins indicated that the low molecular weight proteins dominate the mitochondrial proteome (Fig. 1a), while the distribution of pI values suggested the presence of both membrane proteins and cytosolic proteins (Fig. 1b).

**Fig. 2:**
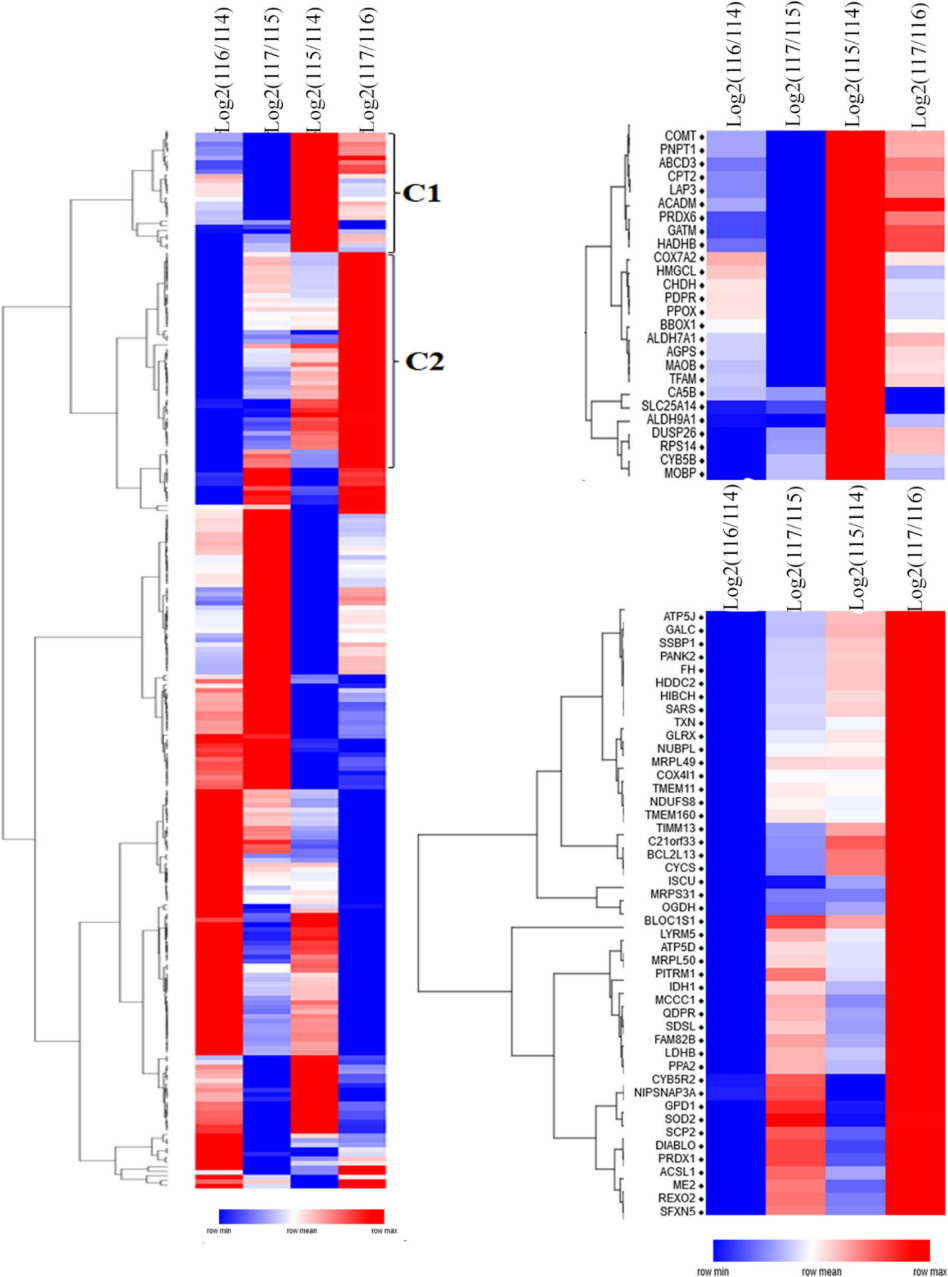
Hierarchical clustering of differentially regulated (+/− 0.3) mitochondrial proteins exhibiting their expressions in early onset AD (log_2_ (115/114)), late onset AD (log_2_ (117/116)) and healthy aging subjects (log_2_ (116/114)). Up-regulated protein expression values are displayed in red, the down-regulation values are in blue, and the intermediate values are in shades of red and blue

To determine the cut-off for up- or down-regulation of proteins, mean iTRAQ ratios of brain mitochondrial proteins between three replicates were measured, then their log fold changes were plotted against log *p*-values in volcano plot (Fig. 1c). Based on the volcano plot and frequency distribution, log_2_ iTRAQ ratio ≤ − 0.30 was set as a down-regulation while ≥ + 0.3 as up-regulation. Of 434 mitochondrial proteins, 208 proteins were differentially regulated. With ±0.3 cut off value, 30/104 proteins were up/down-regulated in the early onset AD, while the corresponding values in the late-onset AD were 40/100. Cellular localization revealed abundances of proteins belonging to the mitochondrion, mitochondrial part, mitochondrial envelope, mitochondrial membrane mitochondrial lumen, mitochondrial matrix, transit peptide: mitochondrion etc. (Fig. 1d). To explore AD-related alterations, the hierarchical clustering of differentially expressed proteins (≥ ± 0.3) was performed (Fig. 2) using Gene Pattern. The mitochondrial proteins clustered under cluster C1 were up-regulated in early-onset AD patients, while proteins of cluster C2 were up-regulated in late AD onset patients.

### Quantitative profile of respiratory complex I

**Fig. 3:**
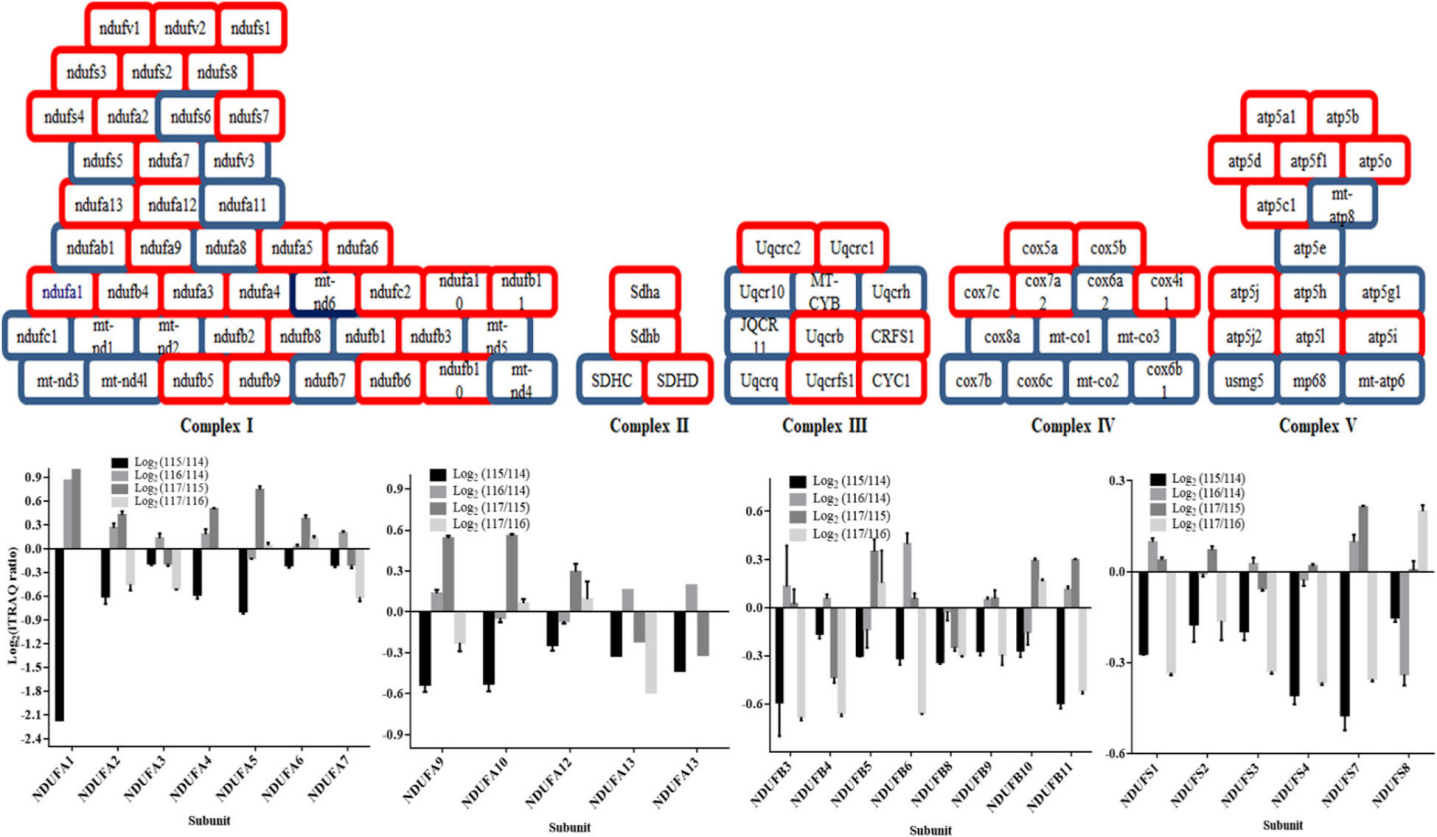
iTRAQ-quantified subunits of complex I. The electron transport chain proteins that were iTRAQ quantified in this study are shown in red color (upper panel). The lower panel show the iTRAQ ratios with standard deviation indicates the abundances and regulation of these mitochondrial subunits in early onset AD (log_2_ (115/114)), late onset AD (log_2_ (117/116)), healthy aging subjects (log_2_ (116/114)) and aging AD (log_2_ (117/115))

**Fig. 4:**
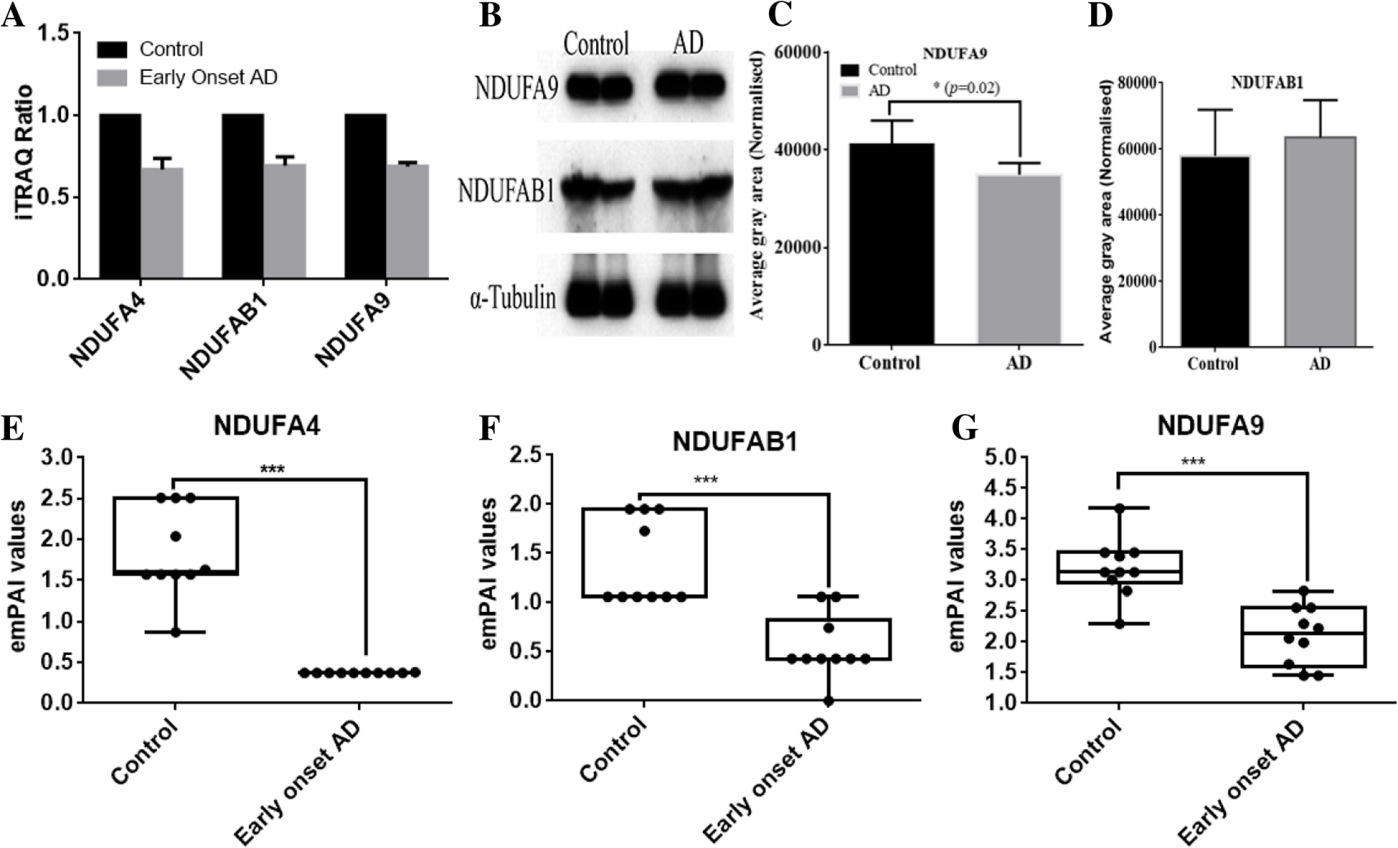
Regulation of NDUFA4, NDUFAB1 and NDUFA9 in early onset AD. **a** iTRAQ quantitative abundances of NDUFA4, NDUFAB1 and NDUFA9 in early onset AD, **b** Western blot analysis of NDUFA9 and NDUFAB1 using mouse monoclonal anti-NDUFA9 and rabbit monoclonal anti-NDUFAB1 that were purchased from Abcam, **c** and **d** Quantitative analysis of immunoblot images by software ImageJ. Differences between means were assessed with the one-way ANOVA test. *P*-value < 0.05 was considered to indicate statistically significant differences. **e** Label free quantitative abundances of subunits NDUFA4, **f** Label free quantitative abundances of subunits NDUFAB1, **g** Label free quantitative abundances of subunits NDUFA9. The dot indicates values in individual brain sample, their biological and technical replicates). Brain mitochondria was isolated, purified using anti-TOM22 magnetic beads and analyzed using Q-Exactive mass spectrometry

**Fig. 5:**
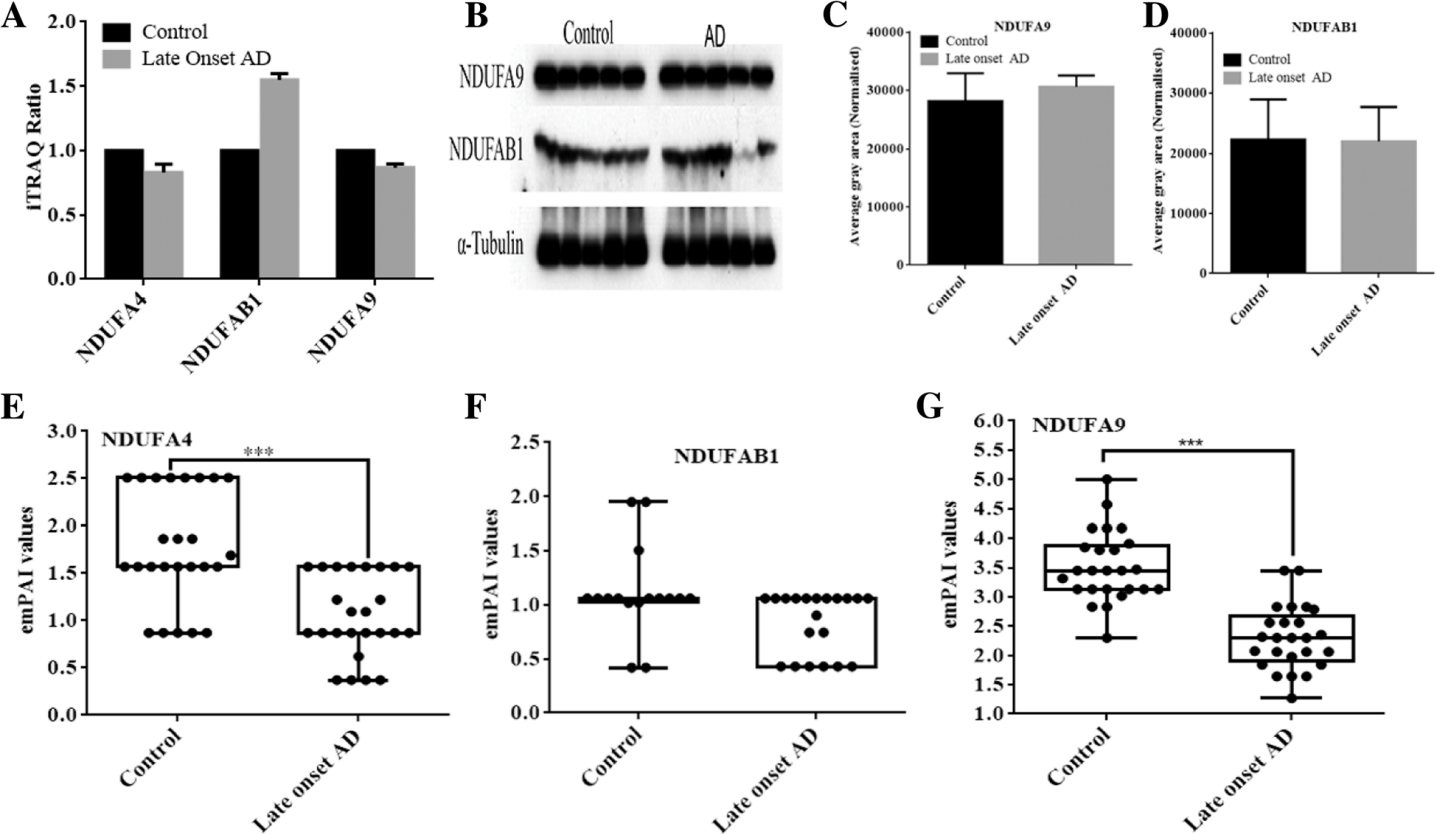
Regulation of NDUFA4, NDUFAB1 and NDUFA9 in late onset AD. **a** iTRAQ quantitative abundances of NDUFA4, NDUFAB1 and NDUFA9 in late onset AD, **b** Western blot analysis of NDUFA9 and NDUFAB1 using mouse monoclonal anti-NDUFA9 and rabbit monoclonal anti-NDUFAB1 in individual samples of control and late onset AD, **c** and **d** Quantitative analysis of immunoblot images by software ImageJ. **e** Label free quantitative abundances of subunits NDUFA4, **f** Label free quantitative abundances of subunits NDUFAB1, **g** Label free quantitative abundances of subunits NDUFA9. The dot indicates values in individual brain sample, their biological and technical replicates. Brain mitochondria was isolated, purified using anti-TOM22 magnetic beads and analyzed using Q-Exactive mass spectrometry

The subunits of the mitochondrial complexes that constitute ETC and ATP-synthase represent a major group of functionally classified mitochondrial proteins. In iTRAQ quantitation, we obtained extensive coverage of these proteins, including 67% of complex I proteins, 75% of complex II, 50% of complex III, 38% of complex IV, and 81% of complex V (Fig. 3, upper panel). The subunits of complex I including NDUFA1, NDUFA2, NDUFA4, NDUFA5, NDUFA9, NDUFA10, NDUFB3, NDUFB6, NDUFB8, NDUFB11, NDUFS4 and NDUFS7 were significantly low abundant in early-onset AD individuals (Fig. 3, lower panel). On the contrary, an abundance of peroxiredoxin-6 and peroxiredoxin-1 that reduces the H_2_O_2_ level and combat oxidative stress were significantly higher in these AD patients (Additional file 1: Table S1). In healthy aging (non-demented, iTRAQ ratio: 116/114) subjects, the abundance of NDUFA1, NDUFA6, and NDUFS8 were altered. The iTRAQ-quantitative brain proteomic data revealed down-regulation of subunits NDUFA2, NDUFA3, NDUFA7, NDUFA13, NDUFB3, NDUFB4, NDUFB6, NDUFB11, NDUFS1, NDUFS3, NDUFS4 and NDUFS7 of complex I in the late-onset AD patients. When an iTRAQ-quantitative abundance of subunits of mitochondrial complex I was compared between early-onset AD individuals, non-demented aging, and late-onset AD patients, we found subunits NDUFA4, NDUFA9, and NDUFAB1 were down-regulated (Fig. 4a) uniquely in the early onset AD individuals. Importantly, these subunits are located at the junction of membrane arm and matrix arm of complex I and their down-regulation indicates possible defect at this junction, particularly, the destabilization of the junction between membrane and matrix arms of complex I. The western blot analysis revealed downregulation of NDUFA9 (Fig. 4b-d). However, to further study the abundances of NDUFA4, NDUFA9, and NDUFAB1, we isolated mitochondria from the brain tissue of each individual patient using the mitochondria isolation kit (MACS Miltenyi Biotec) through Anti-TOM22 MicroBeads. The combined search data of three biological replicates of each individual AD patients is shown in Additional file 4: Table S4. The label-free quantitative profile of mitochondria from each individual patient also revealed down-regulation of NDUFA4, NDUFA9, and NDUFAB1 in the early onset AD individuals (Fig. 4e-g), supporting iTRAQ-quantitative data. However, in late-onset AD, iTRAQ data showed marginal down-regulation of NDUFA4 and NDUFA9 (Fig. 5a), western blot analysis (Fig. 5b-d) did not revealed significant alterations. However, label free proteomics analysis of purified mitochondria showed down regulation of NDUFA4 and NDUFA9 subunits (Fig. 5e-f).

### Quantitative profile of respiratory complexes II-V

**Fig. 6:**
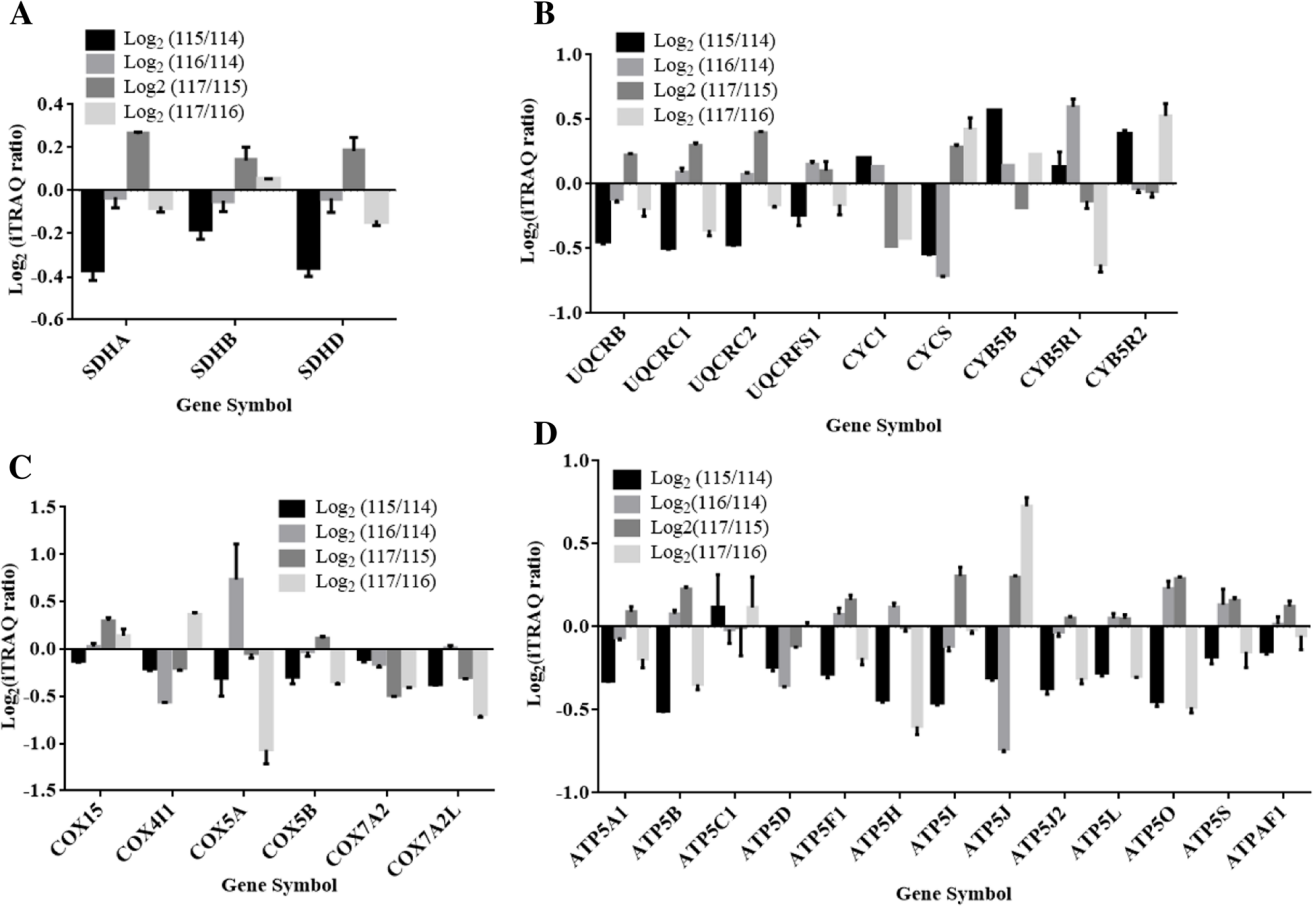
Quantitative abundances of subunits of complex II, III, IV and ATP synthase in brain proteome of early onset AD (log_2_ (115/114)), late onset AD (log_2_ (117/116)) and healthy aging subjects (log_2_ (116/114)). **a** iTRAQ quantitative ratios with standard deviation of quantified subunits of complex II, **b** iTRAQ quantitative ratios with standard deviation of quantified subunits of complex III, **c** iTRAQ quantitative ratios with standard deviation of quantified subunits of complex IV, **d** iTRAQ quantitative ratios of ATP synthase

The subunits of Complex II such as SDHA and SDHD were down-regulated in the early onset AD patients but not affected in healthy aging and late-onset AD patients (Fig. 6a). The components of complex III i.e. UQCRC1, UQCRC2, and UQCRB were down-regulated in the early onset AD (Fig. 6b). The iTRAQ quantitative brain proteome indicated no major alterations in subunits of complex IV (Fig. 6c). The down-regulation of complex III could slow down the oxidation of NADH and FADH_2_, affecting proton gradient across the mitochondrial membrane, which would further affect ATP generation by complex V. The complex V is a multi-subunit enzyme complex consisting of two functional domains, Fo and F1, connected by two stalks. The membrane-embedded Fo domain functions as a proton channel while F1 uses the electrochemical gradient generated by the mitochondrial respiratory chain to convert ADP into ATP. The components of complex V including ATP5B, ATP5H, ATP5I, ATP5J, etc. were down-regulated in the early onset AD (Fig. 6d).

The abundances of peroxiredoxin-6 and peroxiredoxin-1 indicated oxidative stress. Mitochondrial fusion rescues stress by allowing functional mitochondria to complement dysfunctional mitochondria and maintain mitochondrial dynamics [30]. Mitochondrial fusion is mediated by large GTPase proteins such as OPA1, while fission is mediated by dynamin-related protein-1, human fission protein 1 (FIS1), mitochondrial fission factor, and mitochondrial dynamics proteins [31]. In our study, proteins like OPA1, dynamin-like protein, isoform 4 of mitochondrial Rho GTPase 1, mitochondrial fission 1 protein (FIS1) etc. were iTRAQ quantified but their abundances were not significantly altered (Additional file 5: Figure S1a). The Proteins PhB and PhB 2 which function as chaperones for ETC proteins and/or as structural scaffolds, were not altered significantly in the AD and aging Additional file 5: Figure S1a. Mitochondria carry their own genetic material and gene-expression machinery, including ribosomes, which are responsible for synthesizing polypeptides that form essential components of the complexes involved in oxidative phosphorylation (or ATP generation). The quantitative abundances including subunits of large and small 39S were shown in Additional file 5: Figure S1b-c. Abundance of proteins like MRPL49 and MRPL50 was altered in healthy aging.

## Discussion

Here, we report altered brain mitochondriome in AD as discovered by iTRAQ and cross-validated by independent label-free quantitative proteomics experiments using post-mortem brain tissues. Together, the results revealed the altered subunit of complex I, particularly NDUF4 and NDUFA9, suggesting destabilization of the junction between membrane and matrix arm of mitochondria in AD. Of 45 core subunits of complex I, the primary roles of 14 subunits in electron transfer and proton translocation have been established but the exact role of the remaining 31 subunits remains unknown [10]. The functional characterization of individual subunits is needed to gain detailed insight into the function of complex I.

Mitochondria could be an ultimate target for comprehensive proteome analysis because they are centrally involved in a large number of neurodegenerative disorders and aging. We used the iTRAQ proteomic method and quantified 434 mitochondrial proteins in human post-mortem brain tissues, which were comparable to 497 or 531 proteins from mouse liver mitochondrial proteome [32], 615 from normal human heart tissue [16]. Consistent with human heart mitochondrial proteome [16], the molecular weight distribution of human brain mitochondrial proteins showed the dominance of low molecular weight proteins. The integral membrane proteins clustered around pI 8.5–9.0, whereas cytosolic proteins clustered around pI values of 5–6, which was consistent with earlier reports [16, 33].

Complex I is the largest complex constituting about 45 different subunits that oxidize NADH, reduces ubiquinone, and transports protons across the inner membrane, contributing to the proton-motive force [34]. The down-regulation of ETC genes belonging to complexes I, III, IV, and V have been noted in a disease like autism [35], while deficiencies in complex-I activity have been linked to neurodegenerative diseases including AD [7, 9, 36]. Our iTRAQ quantitative proteomics approach revealed significant low abundances of subunits NDUFA1, NDUFA2, NDUFA4, NDUFA5, NDUFA9, NDUFA10, NDUFB3, NDUFB6, NDUFB8, NDUFB11, NDUFS4, NDUFS7 and NDUFAB1 in the early onset AD. The NDUFA1, which was significantly down-regulated in AD patients, encodes the MWFE protein which is essential for the activity of complex I in mammalian mitochondria [37] and its expression level known to affect functional complex I assembly and stability [38]. Human NDUFA1 protein is required in the synthesis of the mtDNA-encoded ND subunits and their incorporation into the complex [39]; whose down-regulation may influence stability and synthesis of the mtDNA-encoded ND subunits in the AD. The NDUFA2 was also down-regulated in AD patients and mutation in this accessory subunit caused reduced activity and neurometabolic disorder [10].

The NDUFA4 that was down-regulated in the early onset AD patients encodes the protein that has NADH dehydrogenase activity and oxidoreductase activity, and its lower abundances have been linked to cytochrome c oxidase deficiency [11]. According to Stroud et al. [40], NDUFA9 is involved in stabilizing the junction between membrane and matrix arms of complex I. NADH-binding site is located on NDUFV1 while NADPH binding site is assigned to NDUFA9 [41]. The subunit NDUFAB1 contains unique attachment site for acyl carrier proteins, and an EF-hand calcium binding domain [42], and was down-regulated in the in the early onset AD patients. Based on the topology of complex I, locations of NDUFA4, NDUFA9, and NDUFAB1 and their down-regulation in iTRAQ quantitation and significantly low abundances in label-free proteomics suggested the destabilized junction between membrane and matrix arms of complex I in the in the early onset AD. Subunits like NDUFA3, NDUFA7, NDUFB4, NDUFS1, and NDUFS3 were uniquely low abundance in late-onset AD patients. The functions of these subunits are still unknown.

Complex II composed of four subunits (SDHA, SDHB, SDHC, and SDHD) and plays a major role in a linear electron transport chain that extends from the flavin and iron-sulfur redox cofactors to the quinone and heme cofactors [43]. SDHA and SDHD were marginally down-regulated in the early onset AD patients and their abundance remains unaffected in the late-onset AD group. The UQCRC1 and UQCRC2 are part of complex III of the mitochondrial RC which mediate formation of the complex between cytochromes c and c1, and assembly of the complex III was down-regulated in the early onset AD patients. In native state, the complex III monomer is quickly converted into a catalytically active homodimer that is incorporated into a supercomplex (respirasome) with complexes I and IV, and this respirasome functions as a single enzyme [44]. However, defects in complex I may affect the function of complex III. COX is the terminal enzyme of the mitochondrial respiratory chain that couples the transfer of electrons from cytochrome c to molecular oxygen and contributes to a proton electrochemical gradient across the inner mitochondrial membrane required for ATP synthesis. The COX5A, COX5B COX7A2, COX7A2L, and CYC1 were down-regulated in late-onset AD patients, which may result in depleted energy production.

Kidney function declines with advancing age where mitochondria have been implicated, for example, mitochondrial functional studies in 6- and 24-month-old rats kidney revealed 37% decrease in cytochrome c activity in 24-month-old rats kidney, linking cytochrome c activity with aging [45]. Similarly, our study also found significantly low abundances of cytochrome C in healthy aging subjects. During studying effects of aging on cytochrome c oxidase activity and other bioenergetic processes such as oxygen consumption, membrane potential and ROS production in rat brain mitochondria, Petrosillo et al. [46] noted the age-dependent decline in the cytochrome c oxidase activity with parallel changes in respiration, membrane potential and an increase in H_2_O_2_ generation.

A major limitation of our study was the low number of patients in the group. However, given the current paucity of proteomics data in brain mitochondria, this report provides a proteomic composition of different subunits of mitochondrial complexes which could be useful for another researcher for a targeted approach. Further, brain mitochondrial proteome revealed alterations in different subunits of each mitochondrial complex, which may add to current scientific knowledge and will guide future efforts to develop novel developments in understanding the disease mechanism and design therapeutic strategies.

In conclusion, this study found destabilized junction between membrane and matrix arms of complex I in the early onset AD. The iTRAQ quantitation of mitochondrial proteome revealed significantly altered human brain mitochondrial proteome in the AD and explored differences in healthy aging and pathophysiology of the age-dependent AD. The proteomics data revealed a significant decline in the cytochrome c oxidase activity in aged human mitochondria brain proteome. The limitation of this remains the low number of patients in the group and it demands more detailed further study in large cohorts.

## Methods

### Brain tissue samples and preparation

Frozen brain samples of the medial frontal gyrus from AD patients and age-matched controls were obtained from Netherlands Brain Bank, Meibergdreef 47, 1105 BA Amsterdam, The Netherlands. The scientific use of the human material was conducted in accordance with the Declaration of Helsinki, and informed consent was obtained from all patients or the guardians of the patients prior to donation of brain tissues upon their death. All procedures were approved and performed in accordance with the ethical guidelines of the Nanyang Technological University ethics board. The medial frontal gyrus of the human brain plays a major role in memory retrieval and the ability to remember previously experienced items/events. The medial frontal gyrus is involved during executive and volitional functions receive and integrate input from multiple systems, and play major role in executive mechanisms operative [47].

Frozen individual brain samples were thawed on ice and equal quantity was pooled in a group-wise manner. The individual clinical details of the patients are listed in Table 1. The brain tissue samples from each patient and control (in equal quantity) were pooled together within the group. We pooled two brain tissues in early onset AD and their age-matched control while old onset AD and control include five-tissue specimen. The tissue samples were homogenized in a urea-acetate buffer (8M urea, 100 mM ammonium acetate, pH 6.0) to prevent asparagine deamidation. The cell debris was removed by centrifugation at 5000 x g for 7 min at 4°C. The proteins were purified by acetone precipitation and 200 μg proteins from each group were loaded into 12.5% SDS-PAGE and run at 80V for 45 min. Each sample lane was excised separately, cut into small pieces (approximately 1 mm^2^ pieces) and washed with 75% acetonitrile (ACN) containing triethylammonium bicarbonate (TEAB, 25 mM). The gel pieces were reduced with Tris 2- carboxyethyl phosphine hydrochloride (5 mM) and then alkylated with methyl methanethiosulfonate (10 mM). Gel pieces were dehydrated using ACN and subjected to protein digestion with sequencing grade modified trypsin (Promega, Madison, WI) at 37°C. The peptides were extracted using 50% acetonitrile, 5% acetic acid and concentrated using vacuum concentrators (Eppendorf AG, Hamburg, Germany).

### iTRAQ labeling and LC-MS/MS analysis

The iTRAQ labeling of dried peptides from both the control and disease groups was performed using a 4-plex iTRAQ reagent Multiplex kit (Applied Biosystems, Foster City, CA) as described earlier [48–51]. The iTRAQ labeling was as follow 114: the Non-demented women brain of 67 ± 3.0-year-old (control); 115: the AD women brain of 68 ± 2.0-year-old, 116: Non-demented women brain of 84 ± 3.2 year old, and 117: AD women brain of 83.8 ± 3.5 year old. The label used for each group is listed in Table 1. iTRAQ experiment was performed in triplicate. Following labeling, the reaction was quenched; labeled peptides were mixed together and dried. Then iTRAQ-labeled peptides were desalted using Sep-Pak C18 cartridges, vacuum dried and fractionated on a C18 column (4.6 × 200 mm, 5 μm, 300 Å) (PolyLC, Columbia, MD, USA) at a flow rate of 1.0 ml/min using HPLC. The mobile phases consisting buffer A (0.02% NH_4_OH in water) and buffer B (0.02% NH_4_OH in 80% ACN) were used to establish 60-min gradient as 97% buffer A for 3 min, 3–10% buffer B for 2 min, 10–35% buffer B for 40 min, 35–70% buffer B for 5 min and 100% buffer B for 10 min at 1 ml/min flow rate. HPLC chromatograms were recorded at 280 nm and sixty fractions were collected using an automated fraction collector. The collected fractions were pooled according to concatenate pooling method, concentrated using vacuum centrifuge, and reconstituted in 0.1% formic acid for LC-MS/MS analysis.

The LC-MS/MS analysis was performed using the Dionex Ultimate 3000 RSLC nanoLC system coupled to a Q-Exactive apparatus (Thermo Fisher, MA). 5μl sample was injected into an acclaim peptide trap column via the autosampler of the Dionex RSLC nanoLC system. Mobile phase A (0.1% FA in 5% ACN) and mobile phase B (0.1% FA in ACN) were used to establish a 60 min gradient. The flow rate was maintained at 300 nl/min. Peptides were analyzed on a Dionex EASY-spray column (PepMap® C18, 3um, 100A) using an EASY nanospray source at an electrospray potential of 1.5 kV. MS scan (350–1600 m/z range) was acquired at a resolution of 70,000 at m/z 200, with a maximum ion accumulation time of 100 ms. Dynamic exclusion was set to 30 s. Resolution for MS/MS spectra was set to 35,000 at m/z 200. The AGC setting was 1E6 for the full MS scan and 2E5 for the MS2 scan. The 10 most intense ions above a 1000 count threshold were selected for HCD fragmentation, with a maximum ion accumulation time of 120 ms. An isolation width of 2 Da was used for the MS2 scan. Single and unassigned charged ions were excluded from MS/MS. For HCD, normalized collision energy was set to 28. The underfill ratio was defined as 0.1%.

### Mitochondria isolation by anti-TOM22 magnetic beads and label free proteomics

Brain tissue lysates were prepared using the Mitochondria Extraction Kit (Miltenyi Biotec, Bergisch Gladbach, Germany) according to the manufacturer’s instructions. In brief, 100 mg brain tissues were cut into small pieces and digested with extraction Buffer. After a centrifugation at 300×g for 5 min at 4°C, the pellet was re-suspended in buffer supplemented with protease inhibitor cocktail and was quickly homogenized using the Gentle MACS Dissociator (Miltenyi Biotec). Mitochondria were magnetically enriched by labeling with 50 μl of anti-TOM22 MicroBeads in separation buffer for 1 h at 4°C. The suspension was then loaded onto an LS column (Miltenyi Biotec) in a magnetic field, which had been placed in a MACS Separator. After washing the column with separation buffer, it was removed from the MACS separator and the magnetically labeled mitochondria were eluted with 1.5 ml separation buffer, centrifuged at 13,000 x g for 2 min, and the pellet was suspended in storage buffer. Before further analysis, the eluates were centrifuged at 12,000 g for 3 min at 4°C. The mitochondrial pellet was re-suspended in PBS buffer. 40μg of protein was loaded to SDS-PAGE and run at 100v for 60 min. Samples were digested and LC-MS/MS analysis was performed according to our earlier protocol [52].

### Mass spectrometric data analysis

Acquired data was further processed using Proteome Discoverer (PD) v1.4 (Thermo Scientific, San Jose, USA) with deisotope and deconvolution in MS/MS. The raw files were directly imported into the PD and further processed using designed workflow. Briefly, this workflow includes five processing nodes numbered from 0 to 5. Node 0 named “spectrum file” allows selecting raw files, node 1 labeled as “spectrum selector” extracts, deisotopes, and deconvolutes the spectra within a retention time window and precursor ion mass window. Node 2 selected search engine SequestHT and Node 3 used Mascot with database search parameters. The parameters set were enzyme: trypsin, maximum miss cleavage: 2, minimum peptide length: 6, maximum peptide length: 144, precursor mass tolerance: 10 ppm, fragment mass tolerance: 0.02 Da, dynamic modification: deamidation of Q and N. Node 5 called “percolator” where target FDR (strict) was set as 0.01, target FDR (relaxed) was set as 0.05. Node 4 named reporter ions quantifier, where the parameter set were Quantification method: iTRAQ 4Plex, peak integration tolerance: 10 ppm, integration method: most confident centroid, mass analyzer: FTMS, MS order: MS2, activation type: HCD, minimum collision energy; 0 and maximum collision energy: 100. Database search was performed against the UniProt human database (sequence 88,473, downloaded on April 2015). The obtained peptide/protein list was exported to Microsoft Excel or processed using an in-house script for further analysis. These proteins were further subjected to MitoCarta database [9] to find the mitochondrial origin. Online Gene Pattern software (http://genepattern.broadinstitute.org) was used for the hierarchical clustering.

### Western blot analysis

Brain proteins were separated on 15% polyacrylamide gels, transferred onto 0.45-μm nitrocellulose membrane (BioRad, Hercules, CA), blocked and probed overnight at 4 °C with the primary antibodies. Monoclonal Antibodies against NDUFA9 and NDUFAB1 (Abcam) were used in this study. ImageJ software used to analyze western blot images.

## Additional files

Additional file 1: **Table S1**. iTRAQ quantified human brain proteome of AD individuals and their age-matched control. The data is combined search of three biological replicates. (XLSX 959 kb)

Additional file 2: **Table S2**. iTRAQ quantified human brain proteome of AD individuals and their age-matched control with more than 2 peptides. The data is combined search of three biological replicates. (XLSX 1485 kb)

Additional file 3: **Table S3**. iTRAQ-quantified Mitochondrial proteins with log2 iTRAQ ratio. (XLSX 107 kb)

Additional file 4: **Table S4**. Proteins identified in purified mitochondria from each individuals. The data presented is the combined search of three biological replicates (XLS 1701 kb)

Additional file 5: **Figure S1**. Quantitative abundances of the proteins involved in mitochondrial fusion and fission and mitoribosomes in brain proteomes of early onset AD (log_2_ (115/114)), late onset AD (log_2_ (117/116)) and healthy aging subjects (log_2_ (116/114)). A) iTRAQ quantitative ratios of proteins involved in mitochondrial fusion and fission, B) and C) iTRAQ quantitative proteins of mitoribosomes. (DOCX 144 kb)

## Abbreviations

AD: Alzheimer’s disease
ALS: Amyotrophic lateral sclerosis
ERLIC: Electrostatic repulsion-hydrophilic interaction chromatography
ETC: Electron transport chain
HD: Huntington’s disease
iTRAQ: Isobaric tag for relative and absolute quantitation
LC-MS/MS: Liquid chromatography-tandem mass spectrometry
OXPHOS: Oxidative phosphorylation
PD: Parkinson’s disease
RC: Respiratory complexes
ROS: Reactive oxygen species
