# Supplementary material for: Quantitative profiling brain proteomes revealed mitochondrial dysfunction in Alzheimer’s disease
Source: Mol Brain. 2019 Jan 28;12:8. doi: 10.1186/s13041-019-0430-y (PMC6350377; doi:10.1186/s13041-019-0430-y)
Supplement: Supplementary file 5 — Figure S1. Quantitative abundances of the proteins involved in mitochondrial fusion and fission and mitoribosomes in brain proteomes of early onset AD (log2 (115/114)), late onset AD (log2 (117/116)) and healthy aging subjects (log2 (116/114)). A) iTRAQ quantitative ratios of proteins involved in mitochondrial fusion and fission, B) and C) iTRAQ quantitative proteins of mitoribosomes. (DOCX 144 kb) [file 13041_2019_430_MOESM5_ESM.docx]

**Quantitative Profiling Brain Proteomes Revealed Mitochondrial Dysfunction in Alzheimer’s Disease**

Sunil S Adav*, Jung Eun Park, and Siu Kwan Sze*


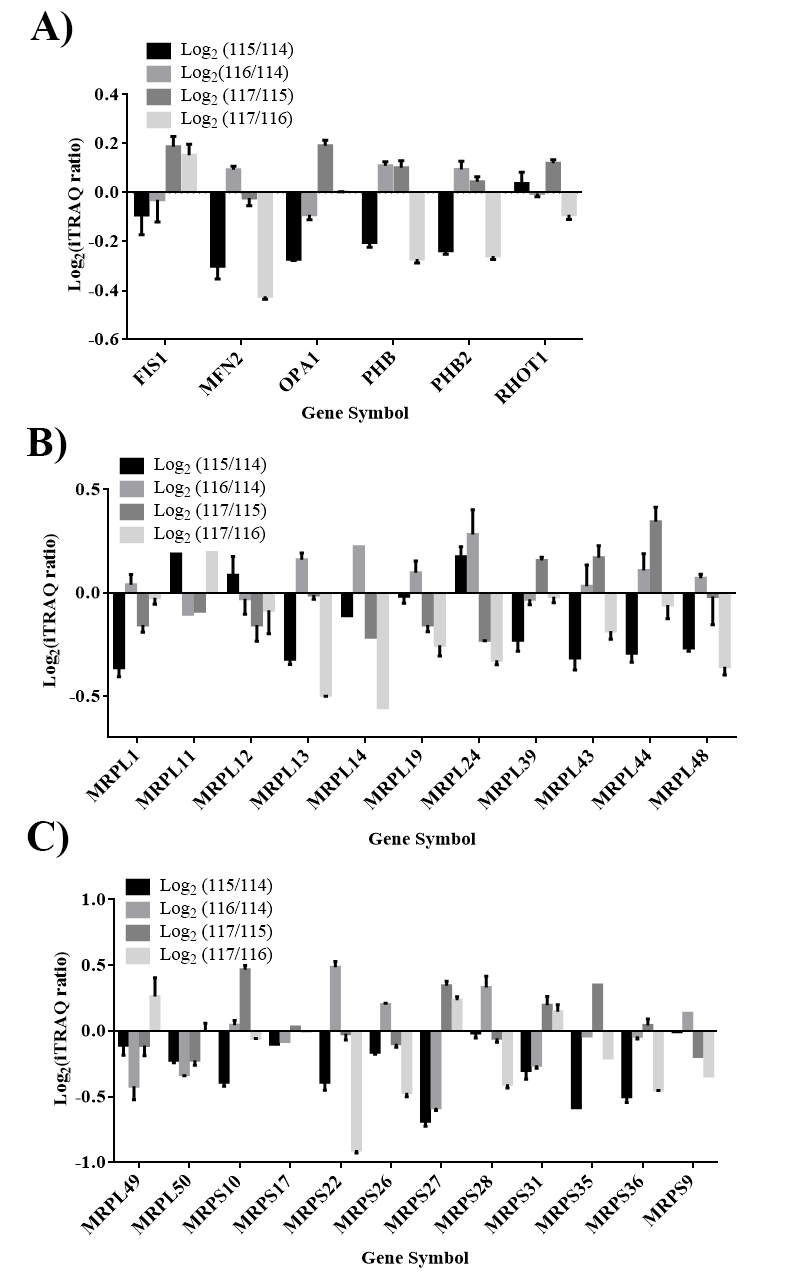


Figure S1. Quantitative abundances of the proteins involved in mitochondrial fusion and fission and mitoribosomes in brain proteomes of early onset AD (log_2_ (115/114)), late onset AD (log_2_ (117/116)) and healthy aging subjects (log_2_ (116/114)). A) iTRAQ quantitative ratios of proteins involved in mitochondrial fusion and fission, B) and C) iTRAQ quantitative proteins of mitoribosomes.
